# Association between skin autofluorescence and coronary calcification in the general population

**DOI:** 10.1371/journal.pone.0309059

**Published:** 2024-08-26

**Authors:** Henderikus E. Boersma, Congying Xia, Melanie M. van der Klauw, Marleen Vonder, Matthijs Oudkerk, Pim van der Harst, Gert Jan Pelgrim, Bruce H. R. Wolffenbuttel, Andries J. Smit, Rozemarijn Vliegenthart

**Affiliations:** 1 Department of Endocrinology, University of Groningen, University Medical Center Groningen, Groningen, The Netherlands; 2 Department of Internal Medicine, University of Groningen, University Medical Center Groningen, Groningen, The Netherlands; 3 Department of Radiology, University of Groningen, University Medical Center Groningen, Groningen, The Netherlands; 4 Department of Epidemiology, University of Groningen, University Medical Center Groningen, Groningen, The Netherlands; 5 Institute for Diagnostic Accuracy B.V, Groningen, The Netherlands; 6 Faculty of Medical Sciences, University of Groningen, Groningen, The Netherlands; 7 Department of Cardiology, University of Groningen, University Medical Center Groningen, Groningen, The Netherlands; Scuola Superiore Sant’Anna, ITALY

## Abstract

**Objective:**

To address the relationship between tissue accumulation of advanced glycation end-products, assessed by skin autofluorescence (SAF), and subclinical atherosclerosis quantified with coronary artery calcium score (CACS) in the general Dutch population.

**Methods:**

A total of 3,839 participants of the LifeLines Cohort Study without diabetes or cardiovascular disease were included in this cross-sectional evaluation. They underwent SAF measurement and cardiac computed tomography to measure CACS. Associations between SAF and CACS was assessed using regression models. Participants at elevated risk for cardiovascular disease were selected by either CACS≥100, or SAF value in the top 15%; overlap and cardiovascular risk profile of these participants were compared.

**Results:**

In univariate analysis, every 1 arbitrary unit (AU) increase in SAF resulted in an odds ratio of 2.91 (95% confidence interval 2.44–3.48, p<0.001) for coronary calcification. After adjustment for cardiovascular risk factors, there was still 20% higher odds of coronary calcification with 1 AU increase in SAF, but significance was lost. In total, 1025 (27%) participants either had high SAF and/or high CACS, of these 441 (12%) had only high SAF, 450 (12%) had only high CACS and 134 (3%) participants had high SAF and high CACS.

**Conclusion:**

In a population-based Dutch cohort, SAF was associated with the degree of coronary calcification. This association was largely explained by classical cardiovascular risk factors. Limited overlap was found in subgroups with high SAF or high CACS, indicating that SAF and CACS may have complementary role in identifying individuals at elevated cardiovascular risk.

## Introduction

Advanced glycation end products (AGEs) play a pre-eminent role in atherosclerosis and cardiovascular disease (CVD) [1]. Vascular complications in diabetes and accelerated atherosclerosis in renal failure are attributed to increased AGE formation in these disorders [2,3]. The evidence of the role of AGEs in atherosclerosis beyond these conditions is growing. The mechanism of AGE accumulation in the absence of diabetes is either by slow accumulation as part of the ageing process or by a receptor mediated process through interaction with the receptor for AGEs (RAGE). AGEs have been found in aortic plaques, and blocking RAGE has been found to reduce atherosclerosis in a murine model [4].

Skin auto-fluorescence (SAF), as measured by the AGE Reader, is a non-invasive technique to quantify the degree of fluorescent AGE accumulation in the skin. This technique has been validated and is strongly associated with AGEs in skin biopsies [5]. SAF predicts cardiovascular complications in diabetes and kidney disease, as well as the progression of atherosclerosis in peripheral arterial disease [6]. SAF is consistently associated with future cardiovascular events and all-cause mortality, also in the general population [7].

The coronary artery calcium score (CACS) is a non-invasive imaging biomarker for CVD based on non-contrast cardiac CT. The CACS reflects the total amount of coronary atherosclerosis. CACS has been found to be a very strong predictor of cardiovascular events in multiple population-based studies, stronger than classical cardiovascular risk factors and other nonclassical risk factors [8]. Using CACS in addition to classical risk factor assessment results in more accurate risk classification in individuals estimated at intermediate risk for CVD [9].

CACS has its role in cardiovascular risk assessment, for SAF there is no clear role yet. AGEs are involved in the atherosclerotic process, but the relationship between SAF and CACS has not been clarified yet. Knowledge about their relationship may help to optimize the cardiovascular risk assessment strategy. In the past studies that investigated the association between SAF and CACS were performed in selected populations with small sample size [10,11]. Recently a study in the general population showed a significant association between SAF and CACS. However, in this study it was not investigated whether SAF and CACS select the same individuals at risk for CVD [12].

Therefore, the primary goal of this study was to address the relationship between tissue accumulation of AGEs, assessed by SAF, and subclinical atherosclerosis quantified with CACS. We further compared participants selected by either high SAF and/or high CACS.

## Materials and methods

### 2.1 Study population and setting

Participants of this study are part of the LifeLines Cohort [13]. The Lifelines cohort study was designed to examine the complex interactions between environmental and genetic factors in healthy ageing and the development of chronic illnesses. From 2006, inhabitants from the northern part of the Netherlands as well as their families were invited by the general practitioner to take part in the Lifelines cohort study. Baseline data have been collected from more than 167,000 participants and was completed in 2013. Every five years, follow-up visits are scheduled. The second-round of assessments started after completion of the baseline assessments and were completed in 2017. The Imaging in Lifelines (ImaLife) study started in August 2017 and its aim is to establish reference values of imaging biomarkers of early stages of coronary artery disease, lung cancer and chronic obstructive pulmonary disease. Participants, aged 45 years and older, who had completed the second-round assessments were invited for computed tomography (CT) examination of the heart and lungs. The detailed in- and exclusion criteria were described previously [14]. For the current analyses, participants of whom SAF measurements were available at baseline and had completed the CT scan were included. Participants of whom cardiac CT images revealed coronary interventions (coronary artery stents or coronary artery bypass grafts) or with known diabetes and/or prior CVD were excluded (n = 162). Prior CVD was defined as self-reported history of myocardial infarction and/or stroke. This resulted in 3839 individuals with data available for analyses.

All individuals provided written informed consent for participation in the Lifelines study. The Lifelines Cohort study was approved by the Medical Ethics Review Committee of the University Medical Center Groningen, document number METC UMCG METc 2007/152.

For the ImaLife study, the individuals provided separate informed consent; the ImaLife study obtained separate approval from the Medical Ethics Review Committee.

### 2.2 Clinical examination

For all clinical examinations, baseline data were used for the current analysis. Medical history, health status and smoking status were collected by self-administered questionnaires. The use of medication was recoded according to the Anatomical Therapeutic Chemical Classification System. Smoking status was classified into never, former and current smoking [15]. Body weight, height and waist circumference without shoes and heavy clothing were measured by trained researchers. Body weight was rounded to the nearest 0.1kg, while body height and waist circumference were rounded to the nearest 0.5cm. Body mass index (BMI) was calculated based on body weight and height with the formula: BMI = kg/m^2^. Heart rate and systolic and diastolic blood pressure (BP) were measured using the automated Dinamap monitor (GE Healthcare, Freiburg, Germany) ten times during 10 min. Values of BP and heart rate were registered by averaging the final three readings. All data is pseudonymised to guarantee the participants’ privacy.

### 2.3 Biochemical measurements

For all biochemical measurements, baseline data were used for this study. Participants underwent blood sampling in the fasting state between 08:00 and 10:00 hours; the blood was transported to the central Lifelines laboratory facility. Hexokinase method was used to measure fasting blood glucose. On the same day of collection, EDTA-anticoagulated blood was used on the Cobas Integra 800 CTS analyser (Roche Diagnostics Nederland, Almere, the Netherlands) to measure HbA_1c_ with National Glycohemoglobin Standardization Program (NGSP) certified turbidimetric inhibition immunoassay method. Total cholesterol, high-density lipoprotein (HDL) cholesterol, triacylglycerol, low-density lipoprotein (LDL) cholesterol and serum creatinine, were measured on a Roche Modular P chemistry analyser (Roche, Basel, Switzerland) with corresponding methods [15]. Renal function was evaluated by calculating the estimated (e)GFR with Chronic Kidney Disease Epidemiology Collaboration (CKD-EPI) validated formula [16].

### 2.4 Skin autofluorescence

SAF was measured at baseline using the AGE Reader (DiagnOptics Technologies BV, Groningen, the Netherlands), as described previously [5,17]. Measurements were performed at the volar side of the forearm, 10cm below the elbow fold, at room temperature. The AGE Reader illuminates the skin surface, with an excitation light source of which wavelength is between 300 and 420nm with a peak intensity at ~370nm, and is guarded against surrounding light. Emission light (wavelength of 420-600nm) and reflected light (wavelength of 300-420nm) from the skin is measured with an internal spectrometer. The emitted light is divided by the reflected light and multiplied by 100, expressed as arbitrary units (AU). This results in the SAF measurement. Sex-stratified calculation of age-adjusted SAF z scores was conducted based on the total Lifelines cohort.

### 2.5 Coronary artery calcium score

Participants underwent an ECG-gated non-enhanced cardiac CT scan using third-generation dual-source CT (Somaton Force, Siemens Healthineers, Germany). The entire heart from the carina to the apex was scanned. Imaging acquisition settings included prospective ECG triggering at 65% R-R interval, tube voltage 120 kVp, reference tube current 64 mAs, field of view of 250mm, slice thickness and increment of 3.0 mm and 1.5 mm, filtered-back projection and reconstruction kernel Qr36. Images were analysed and Agatston score was determined by a well-trained researcher using dedicated commercial software (Syngo.via VB30A, CaScoring, Siemens Healthineers, Germany). Participants were stratified into four common CACS risk categories based on Agatston score: 0, 1–99, 100–399, ≥400 [18].

### 2.6 Calculations, definitions and statistical analyses

Data are presented as mean ± SD. If the data were not normally distributed, median and interquartile range (IQR) are presented. Means were compared between groups with analysis of variance (ANOVA). Medians were compared using the Mann–Whitney *U* test. The *χ*^2^ test was used to compare categorical variables. Using the ACC/AHA Pooled Cohort equations [19] the 10-year risk of atherosclerotic cardiovascular disease (ASVCD) was calculated, based on age, current smoking, systolic BP, treatment of hypertension, total and HDL cholesterol levels, and history of diabetes mellitus (no in this case, since individuals with known diabetes were excluded for the current study). Univariate linear regression analyses were performed to assess the association of cardiovascular risk factors with skin autofluorescence and the amount of coronary calcification. Because of skewed distribution, logarithmic transformation was conducted for CACS by the formula ln(CACS+1). To examine the association between SAF and the presence of coronary calcification, several logistic regression models were then developed. Univariate logistic regression was first conducted (model 1), followed by a multivariate modelling adjustment for age and sex (model 2). Additional adjustments for smoking status, total cholesterol, HDL cholesterol, systolic blood pressure was conducted (model 3) to examine whether the relationship between SAF and presence of coronary calcification was independent of classical cardiovascular risk factors. Sensitivity analysis was conducted by adjusting for integrated 10-year ASVCD risk scores (model 4). Individuals missing information on any covariate were excluded from each analysis, for model 2 and 3 no single covariate had more than 3 missing (0,1%). ASCVD risk score could be calculated for 97,8% of participants, missing 89 individuals for analysis.

Furthermore, the difference between CACS based and SAF based individual risk assessment was investigated. We used CACS ≥100 as a cut-off to define individuals who had elevated CVD risk. This resulted in 584 participants with CACS ≥100. Absolute cut-off values for SAF are not available. Since around 15% of participants were selected with the cut-off value of CACS ≥100, we selected the top 15% participants with highest SAF values, this resulted in a cut-off value of 2.35 AU. All analyses were conducted using PASW Statistics (Version 25, IBM, Armonk, NY, USA). Values of *p*<0.05 were considered statistically significant.

## Results

Table 1 provides the clinical characteristics of the study population stratified by the degree of coronary calcification. Fifty-three percent of the study population had no coronary calcification (CACS = 0). Age, sex, BMI, systolic and diastolic BP, creatinine, cholesterol, glucose and smoking behavior differed between CACS categories. Subjects with higher CACS were older, more likely male and more likely current or former smokers, and had on average higher BMI, systolic and diastolic BP, total cholesterol, fasting glucose and creatinine. With a higher CACS category, more subjects used statins and/or BP-lowering drugs.

**Table 1:** Clinical characteristics of the study population at baseline by coronary artery calcium score subgroups.

Characteristic	CACS 0	CACS 1–99	CACS 100–399	CACS ≥400	P value
Sex (*n*; male/female)	599/1455	643/558	228/145	159/52	P<0.000
Men (%)	29%	54%	61%	75%	P<0.000
Age (years)	44.3±6.1	48.3±7.6	51.0±7.8	55.0±8.2	P<0.000
BMI (kg/m^2^)	25.4±3.7	26.6±3.8	26.7±3.7	26.8±3.5	P<0.000
Systolic BP (mmHg)	124±14	130±14	133±14	134±14	P<0.000
Diastolic BP (mmHg)	74±8	77±9	79±9	80±9	P<0.000
Heart rate (bpm)	72±10	71±10	72±11	71±12	P = 0.160
Creatinine (μmol/l)	72±12	76±12	77±13	78±12	P<0.000
eGFR (ml/min)	96±13	93±12	92±13	91±13	P<0.000
Total cholesterol (mmol/l)	4.9±0.9	5.3±0.9	5.5±1.0	5.5±1.0	P<0.000
HDL-cholesterol (mmol/l)	1.5±0.4	1.4±0.4	1.4±0.4	1.3±3.4	P<0.000
LDL-cholesterol (mmol/l)	3.1±0.8	3.5±0.8	3.7±0.8	3.7±0.9	P<0.000
Triacylglycerol (mmol/l)	1.1±0.7	1.4±0.9	1.5±1.1	1.5±1.2	P<0.000
Glucose (mmol/l)	4.9±0.5	5.0±0.5	5.1±0.5	5.1±0.5	P<0.000
HbA_1c_ (mmol/mol)	36±3	37±3	38±3	37±3	P = 0.775
Never Smoking (%)	49%	43%	31%	25%	P<0.000
Current smoking (%)	17%	19%	25%	25%	P<0.000
Former smoking (%)	34%	38%	44%	50%	P<0.000
% with BP-lowering therapy	5.5%	11.5%	17.4%	23.2%	P<0.000
% with statin use	1.1%	4.4%	5.6%	12.3%	P<0.000

Data are presented as mean ± standard deviation, number or %.

CACS, coronary artery calcium score; BMI, body mass index; BP, blood pressure; HDL, high-density lipoprotein; LDL, low-density lipoprotein.

Missing values for heart rate, systolic and diastolic BP n = 1; Total cholesterol, HDL, LDL and Triacylglycerol n = 3; glucose n = 7; Creatinine and eGFR n = 11; HbA_1c_ n = 16. All other variables had no missing values.

### 3.1 Skin autofluorescence and coronary calcification

**Fig 1:**
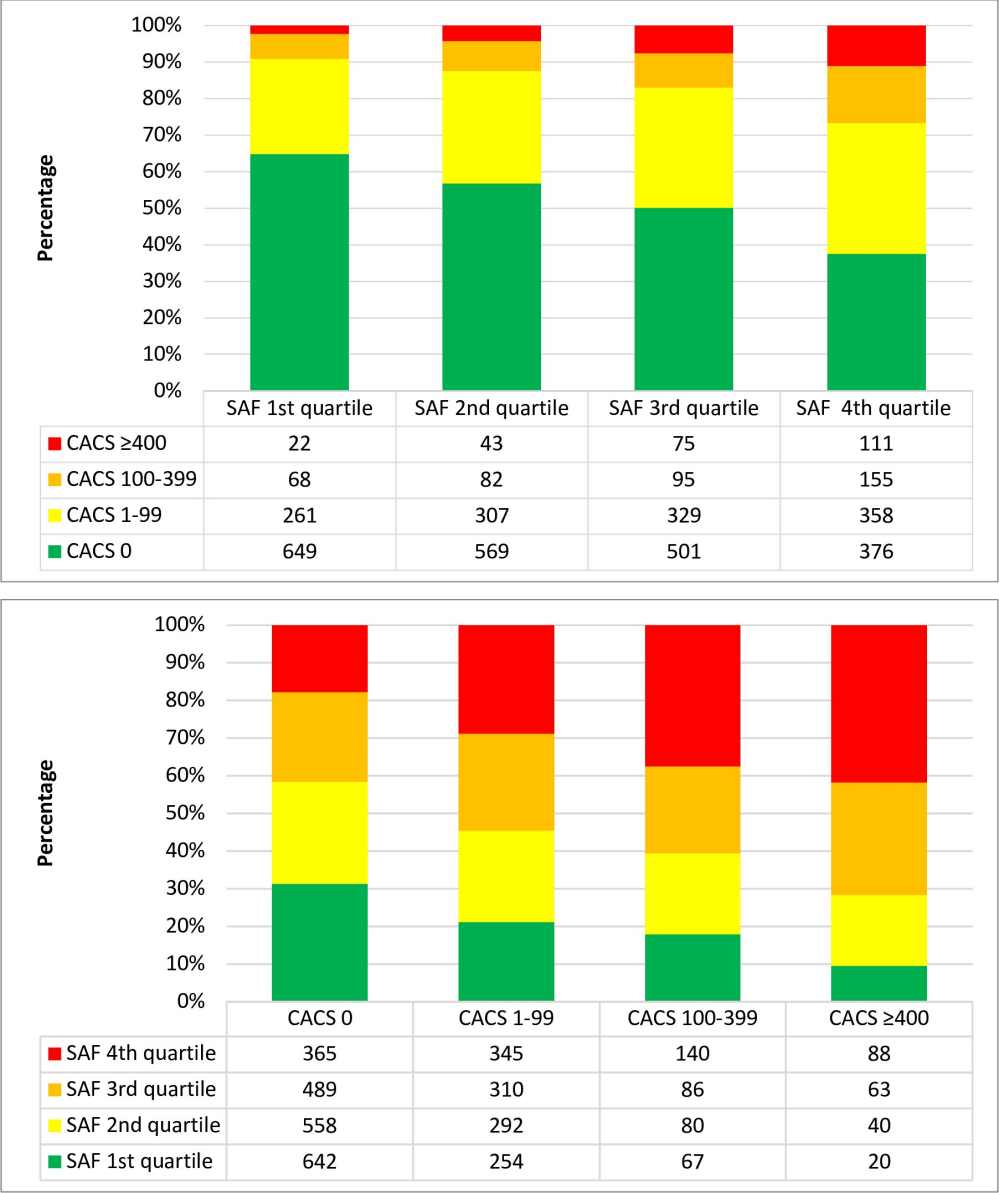
**(top).** Prevalence of degree of CACS in relationship to SAF stratified by quartiles, namely 1st quartile 1.08–1.70AU, 2nd quartile 1.71–1.92AU, 3rd quartile 1.93–2.19AU and 4th quartile 2.20–4.39AU. (bottom). Distribution of quartiles of SAF in relationship to CACS categories, namely CACS = 0, CACS 1–99, CACS 100–399, and CACS ≥400.

Fig 1 (top section) shows the degree of CACS in relationship to quartiles of SAF. In higher quartiles of SAF there was higher degree of CACS. 111 subjects (44%) with CACS ≥400 were in the highest quartile of SAF. Still the largest group in the highest quartile of SAF had CACS of 0 (38%). The bottom section of Fig 1 shows the quartiles of SAF in relationship to CACS categories. Out of the subjects with CACS ≥400 the largest group was in the highest quartile of SAF (42%). Still 18% of participants with CACS of 0 were in the highest quartile of SAF.

### 3.2 Association of skin autofluorescence and coronary calcification

Univariate linear regression showed a significant association between SAF and CACS (β = 0.216 p<0.001, Table 2). Age, BMI, systolic and diastolic BP, total cholesterol, LDL cholesterol, triacylglycerol, fasting glucose, HbA1c, current and former smoking, and the use of BP-lowering drugs and statins were positively associated with SAF and CACS. HDL-cholesterol and eGFR both had significant negative associations with SAF and CACS. Male sex was associated with higher SAF and CACS.

**Table 2:** Univariate linear regression model for skin autofluorescence and coronary artery calcium score in non-diabetic primary prevention group.

Characteristic	Coefficient β Ln(CACS+1)	P value	Coefficient β SAF	P value
Sex (male/female)	-0.278	P<0.000	-0.084	P<0.000
Waist (cm)	0.227	P<0.000	0.095	P<0.000
Age (years)	0.411	P<0.000	0.392	P<0.000
BMI (kg/m^2^)	0.134	P<0.000	0.053	P<0.001
Systolic BP (mmHg)	0.235	P<0.000	0.105	P<0.000
Diastolic BP (mmHg)	0.224	P<0.000	0.065	P<0.000
eGFR (ml/min)	-0.137	P<0.000	-0.191	P<0.000
Total cholesterol (mmol/l)	0.249	P<0.000	0.146	P<0.000
HDL-cholesterol (mmol/l)	-0.156	P<0.000	-0.035	P<0.029
LDL-cholesterol (mmol/l)	0.279	P<0.000	0.145	P<0.000
Triacylglycerol (mmol/l)	0.187	P<0.000	0.067	P<0.000
Glucose (mmol/l)	0.171	P<0.000	0.121	P<0.000
HbA_1c_ (mmol/mol)	0.168	P<0.000	0.161	P<0.000
Current smoking (yes/no)	0.073	P<0.000	0.122	P<0.000
Former smoking (yes/no)	0.093	P<0.000	0.091	P<0.000
BP-lowering therapy (yes/no)	0.188	P<0.000	0.108	P<0.000
Statins (yes/no)	0.160	P<0.000	0.047	P<0.004
Ln(CACS+1)	x	x	0.216	P<0.000
Skin autofluorescence (AU)	0.216	P<0.000	x	x

CACS, coronary artery calcium score; BMI, body mass index; BP, blood pressure; HDL, high-density lipoprotein; LDL, low-density lipoprotein.

Table 3 shows univariate and multivariate logistic regression analyses of SAF and CACS >0, with CACS 0 as the reference group. Every 1 AU increase in SAF was associated with 2.91 (95% CI 2.44–3.48, p<0.001) odds of having coronary calcification. After adjustment for age and sex a significant association between SAF and positive CACS remained (OR 1.36,95% CI 1.11–1.66, p = 0.002). After additional adjustment for cardiovascular risk factors, either as individual parameters (model 3) or as a risk score using ASCVD (model 4), there was still 20% higher odds of coronary artery calcium with a 1 AU increase in SAF, but significance was lost. Stratified by sex we found a significant association between SAF and CACS>0, for men (OR 2.66, 95% CI 2.00–3.58, p<0.001) and for women (OR 2.81, 95% CI 2.23–3.55, p<0,001). After additional adjustment for cardiovascular risk factors, the association was mitigated to an OR of 1.18–1.20, and lost statistical significance (S1 Table).

**Table 3:** Odds ratios for presence of coronary artery calcium by 1 unit increase of skin autofluorescence.

	CACS >0		
	Odds ratio	95%CI	P value
Model 1	2.91	(2.44, 3.48)	p<0.001
Model 2	1.36	(1.11, 1.66)	P = 0.002
Model 3	1.19	(0.97, 1.47)	P = 0.102
Model 4	1.21	(0.96, 1.48)	P = 0.069

CACS: Coronary artery calcium score; CI: Confidence interval.

Reference group: Coronary artery calcium score equals to zero.

Model 1: Univariate logistic regression modelling of skin autofluorescence.

Model 2: Adjusted for age and sex.

Model 3: Model 2 plus additional adjustments for smoking status, total cholesterol, high-density lipoprotein, systolic blood pressure.

Model 4: Alternative adjusting for age, sex and integrated 10-years risk scores of atherosclerotic cardiovascular diseases (ASCVD), as opposed to each cardiovascular risk component.

### 3.3 Subjects selected by SAF and CACS

**Fig 2:**
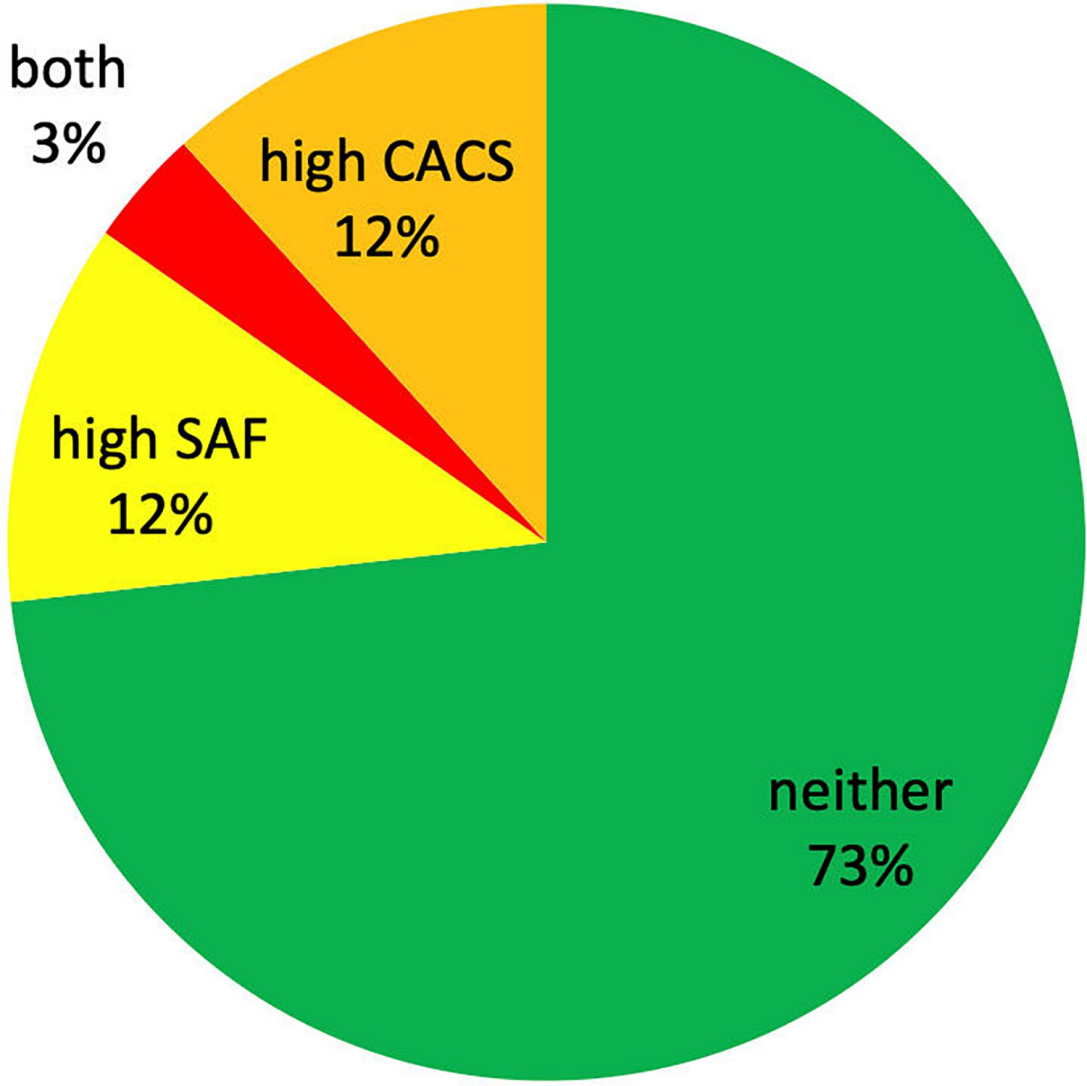
Prevalence of either CACS ≥100 (12%), SAF >2.35 AU (12%) or both.

In total, 1025 (27%) participants either had high SAF and/or high CACS, of these 441 (12%) had only high SAF, 450 (12%) had only high CACS and 134 (3% of the whole populations) participants had both high SAF and high CACS (Fig 2). These 134 subjects comprised 13% of all the selected subjects.

Table 4 shows the characteristics of these different populations. Subjects with both high SAF and CACS had the worst cardiovascular risk profile, having higher age, BMI, systolic BP, more current and former smoking, and higher prevalence of BP-lowering drugs and statins. This resulted in a higher ASCVD risk score and a higher proportion at intermediate risk or higher. Subjects with either high SAF or high CACS had a worse cardiovascular risk profile compared to subjects without high SAF and high CACS. Subjects with only high SAF compared with only high CACS were more likely female and more likely to be current smokers. Those with high CACS compared with those with high SAF had higher LDL-cholesterol, higher systolic-BP, while using more BP-lowering drugs, statins and were more likely male.

**Table 4:** Clinical characteristics of the study population by high SAF, high CACS or both.

Characteristic	Neither	High SAF	High CACS	Both
Sex (*n*; male/female)	1051/1753	191/250	300/150	87/47
Men (%)	37%	43%	67%	65%
Age (years)	45.1 (6.6)	51.0 (7.9)	51.4 (7.8)	56.0 (8.6)
BMI (kg/m^2^)	25.8 (3.8)	26.3 (4.1)	26.7 (3.7)	26.7 (3.6)
Systolic BP (mmHg)	126 (14)	128 (14)	133 (14)	134 (15)
Diastolic BP (mmHg)	75 (9)	76 (9)	80 (9)	78 (9)
eGFR (ml/min)	95 (12)	91 (13)	92 (13)	89 (14)
Total cholesterol (mmol/l)	5.0 (0.9)	5.2 (0.9)	5.5 (1.0)	5.5 (0.9)
HDL-cholesterol (mmol/l)	1.5 (0.4)	1.5 (0.4)	1.4 (0.4)	1.4 (0.4)
LDL-cholesterol (mmol/l)	3.2 (0.8)	3.4 (0.8)	3.7 (0.9)	3.7 (0.8)
Triacylglycerol (mmol/l)	1.2 (0.8)	1.3 (0.8)	1.5 (1.1)	1.5 (1.0)
Glucose (mmol/l)	4.9 (0.5)	5.0 (0.5)	5.1 (0.5)	5.2 (0.6)
HbA_1c_ (mmol/mol)	37 (3)	37 (2)	37 (4)	39 (2)
Current smoking (%)	16.0%	27.0%	22.8%	32.3%
Former smoking (%)	34.7%	40.5%	44.5%	51.1%
Never smoking (%)	49.3%	32.5%	32.7%	16.5%
% with BP-lowering therapy	7.0%	11.8%	19.1%	20.9%
% with statins	2.2%	3.2%	7.6%	9.7%
ASCVD	2.1%	4.4%	5.9%	9.1%
% ASCVD >7.5%	4.7%	16.3%	30.2%	49.3%

Data are presented as mean ± SD, number or %.

CACS, coronary artery calcium score; BMI, body mass index; BP, blood pressure; HDL, high-density lipoprotein; LDL, low-density lipoprotein; ASCVD, 10-years risk scores of atherosclerotic cardiovascular diseases.

## Discussion

In a general population without diabetes and/or CVD, increased AGE accumulation as measured by SAF was associated with coronary calcification. This association was largely explained by classical cardiovascular risk factors. There was limited overlap in selected subjects at elevated risk based on either high CACS or high SAF. Of all individuals with either high CACS or high SAF, only 13% of individuals were selected by both. This suggests that SAF and CACS may have complementary roles in identifying individuals at elevated risk.

AGEs are a heterogeneous group of compounds that are formed during a series of non-enzymatical reactions between sugar and amino groups in proteins, lipids and nucleic acids [20]. Accumulation of AGEs is involved in several pathophysiological pathways, predominantly atherosclerosis-related vascular damage independently and simultaneously with diabetes [1]. Also, the interaction of AGEs with RAGE can induce alterations in coagulation and fibrinolysis, which contributes to thrombogenesis, hypercoagulability, hypofibrinolysis, and consequently result in occlusive thrombosis that is implicated in cardiac ischemia and infarction [20]. CACS is a quantification of coronary calcified lesions that are, if completely calcified, more likely to be stable, but the CACS as such is closely related to the total burden of coronary atherosclerosis [21].

Earlier studies have reported a significant association between AGEs and coronary calcification in smaller cohorts of patients with type 2 diabetes [10] and kidney disease [11]. The study by Pan et al confirmed this association in the general population [12]. In these studies, the association between SAF and CACS remained significant after correction for cardiovascular risk factors. We found a strong association between SAF and coronary calcification presence, with odds ratio of 2.91 in univariate analysis; the association was largely explained by cardiovascular risk factors. We still found 20% higher odds of coronary calcification with a 1 AU increase in SAF after adjusting for cardiovascular risk factors, but the association lost significance. Unlike the study by Pan et al., we have excluded participants with diabetes from analysis. Moreover, our study population was on average 10 years younger. One explanation could be that elevated SAF in patients with diabetes and chronic kidney disease reflects long-term cumulative AGE accumulation due to exposure to metabolic stress over time. This effect of cumulative metabolic stress is less pronounced in a cohort with lower prevalence of cardiovascular risk factors and lower age.

The other key finding is that SAF and CACS mostly select different populations. Of all the selected subjects by either high SAF or CACS, only 13% were selected by both. This is unexpected because both SAF and CACS are strongly associated with future cardiovascular disease and mortality. The value of CACS as a strong cardiovascular risk-predictor has been demonstrated repeatedly [9]. This predictive value has been validated multiple times for different ethnicities, various co-morbidities and age groups. Risk prediction is improved by adding CACS to classical risk factors. Although current guidelines do not yet recommend the usage of CACS as standard in preventive management, its use is considered to improve cardiovascular risk assessment in individuals at intermediate risk. In previous studies high SAF has been a strong predictor of cardiovascular and all-cause mortality in diabetes mellitus, CVD and renal disease [6,22]. Previously, our group reported a threefold increased risk of incident CVD and a fivefold increased risk of all-cause mortality in the general population [7]. This relationship remained statistically significant after correction for confounding factors, including cardiovascular risk factors. In the current study, the limited overlap in selected populations based on high SAF or high CACS indicates that SAF and CACS may reflect the heterogeneity of the atherosclerosis process, and play complementary roles in predicting cardiovascular events and mortality. Interesting to note is the gender difference between high SAF and high CACS, particularly, individuals with only high CACS were more likely to be men, compare to those with only high SAF. This sex difference could be explained by the fact that women develop coronary calcifications on average later in life compared to men. Also myocardial infarction without high burden of coronary calcification is more common in females [23].

Ongoing follow-up in the context of Lifelines will show whether high SAF in addition to high CACS predicts incident CVD. Possible uses of SAF could be to reclassify subjects with high CACS in a higher risk group as well as select another group at risk for CVD not selected by CACS or traditional risk factors. Differences in predicted risk of CVD and mortality between SAF and CACS also need to be investigated.

Strengths of the current study is the large number of participants and sufficient power to investigate the association between SAF and CACS in detail. Limitations of this study also need to be addressed. Since no follow-up data after inclusion in ImaLife are available yet, this study was limited to a cross-sectional analysis. Therefore, a causal inference between SAF and CACS cannot be made. Second, although populations with different cardiovascular profiles were identified as based on high SAF versus high CACS, follow-up data are needed to compare the risk of CVD and mortality between the groups with either high SAF and/or CACS. Third, there is an interval of 4 to 10 years between the SAF testing and CACS measurements, thus the estimated association between SAF and CACS may not be accurate, since SAF values could have increased during the time lag or coronary calcifications could have been formed after the SAF measurement. Furthermore, adjusted covariates for the modelling we used were from the baseline examination, which may have changed over time. This may also lead to a less accurate estimation of the association.

SAF can only be reliably measured in subjects with skin photo type I-IV, or reflectance values above 6% [24]. The vast majority of Lifelines participants have a Caucasian ethnicity (>98%), therefore influence of skin pigmentation differences is very limited. To reduce the impact of seasonal increase of skin pigmentation, SAF measurements are always performed on the volar side of the forearm.

## Conclusions

AGE accumulation as reflected by SAF is associated with the degree of coronary calcification in a population-based Dutch cohort. This association is largely explained by classical cardiovascular risk factors. Secondly, the limited overlap in subgroups based on high SAF or high CACS indicates that SAF and CACS may play different roles in predicting cardiovascular events and mortality, and may have complementary roles in identifying individuals at elevated risk of CVD.

## Supporting information

S1 Table Odds ratios for presence of coronary artery calcium by 1 unit increase of skin autofluorescence by sex. (DOCX)

## Abbreviations

AGEs: advanced glycation end products
RAGE: receptor for advanced glycation end products
SAF: skin auto-fluorescence
CACS: coronary artery calcium score
ImaLife: Imaging in Lifelines
ASVCD: atherosclerotic cardiovascular disease

## References

[1] Fishman SL, Sonmez H, Basman C, Singh V, Poretsky L. The role of advanced glycation end-products in the development of coronary artery disease in patients with and without diabetes mellitus: a review. Mol Med. 2018;24(1):59. doi: 10.1186/s10020-018-0060-3 30470170 PMC6251169

[2] Brownlee M. Glycation products and the pathogenesis of diabetic complications. Diabetes Care. 1992;15(12):1835–43. doi: 10.2337/diacare.15.12.1835 1464241

[3] Busch M, Franke S, Ruster C, Wolf G. Advanced glycation end-products and the kidney. Eur J Clin Invest. 2010;40(8):742–55. doi: 10.1111/j.1365-2362.2010.02317.x 20649640

[4] Del Turco S, Basta G. An update on advanced glycation endproducts and atherosclerosis. Biofactors. 2012;38(4):266–74. doi: 10.1002/biof.1018 22488968

[5] Meerwaldt R, Graaff R, Oomen PHN, Links TP, Jager JJ, Alderson NL, et al. Simple non-invasive assessment of advanced glycation endproduct accumulation. Diabetologia. 2004;47(7):1324–30. doi: 10.1007/s00125-004-1451-2 15243705

[6] Cavero-Redondo I, Soriano-Cano A, Alvarez-Bueno C, Cunha PG, Martinez-Hortelano JA, Garrido-Miguel M, et al. Skin Autofluorescence-Indicated Advanced Glycation End Products as Predictors of Cardiovascular and All-Cause Mortality in High-Risk Subjects: A Systematic Review and Meta-analysis. J Am Heart Assoc. 2018;7(18):e009833. doi: 10.1161/JAHA.118.009833 30371199 PMC6222966

[7] van Waateringe RP, Fokkens BT, Slagter SN, van der Klauw MM, van Vliet-Ostaptchouk JV, Graaff R, et al. Skin autofluorescence predicts incident type 2 diabetes, cardiovascular disease and mortality in the general population. Diabetologia. 2019;62(2):269–80. doi: 10.1007/s00125-018-4769-x 30460578 PMC6323092

[8] Lin JS, Evans CV, Johnson E, Redmond N, Coppola EL, Smith N. Nontraditional Risk Factors in Cardiovascular Disease Risk Assessment: Updated Evidence Report and Systematic Review for the US Preventive Services Task Force. JAMA. 2018;320(3):281–97. doi: 10.1001/jama.2018.4242 29998301 PMC13564044

[9] Greenland P, Blaha MJ, Budoff MJ, Erbel R, Watson KE. Coronary Calcium Score and Cardiovascular Risk. J Am Coll Cardiol. 2018;72(4):434–47. doi: 10.1016/j.jacc.2018.05.027 30025580 PMC6056023

[10] Hangai M, Takebe N, Honma H, Sasaki A, Chida A, Nakano R, et al. Association of Advanced Glycation End Products with coronary Artery Calcification in Japanese Subjects with Type 2 Diabetes as Assessed by Skin Autofluorescence. J Atheroscler Thromb. 2016;23(10):1178–87. doi: 10.5551/jat.30155 26961217 PMC5098918

[11] Wang AY, Wong CK, Yau YY, Wong S, Chan IH, Lam CW. Skin autofluorescence associates with vascular calcification in chronic kidney disease. Arterioscler Thromb Vasc Biol. 2014;34(8):1784–90. doi: 10.1161/ATVBAHA.114.303378 24876353

[12] Pan J, Bao X, Goncalves I, Jujic A, Engstrom G. Skin autofluorescence, a measure of tissue accumulation of advanced glycation end products, is associated with subclinical atherosclerosis in coronary and carotid arteries. Atherosclerosis. 2022;345:26–32. doi: 10.1016/j.atherosclerosis.2022.02.014 35196628

[13] Stolk RP, Rosmalen JG, Postma DS, de Boer RA, Navis G, Slaets JP, et al. Universal risk factors for multifactorial diseases: LifeLines: a three-generation population-based study. Eur J Epidemiol. 2008;23(1):67–74. doi: 10.1007/s10654-007-9204-4 18075776

[14] Xia C, Rook M, Pelgrim GJ, Sidorenkov G, Wisselink HJ, van Bolhuis JN, et al. Early imaging biomarkers of lung cancer, COPD and coronary artery disease in the general population: rationale and design of the ImaLife (Imaging in Lifelines) Study. Eur J Epidemiol. 2020;35(1):75–86. doi: 10.1007/s10654-019-00519-0 31016436 PMC7058676

[15] van Waateringe RP, Slagter SN, van der Klauw MM, van Vliet-Ostaptchouk JV, Graaff R, Paterson AD, et al. Lifestyle and clinical determinants of skin autofluorescence in a population-based cohort study. Eur J Clin Invest. 2016;46(5):481–90. doi: 10.1111/eci.12627 27002914 PMC5111733

[16] Levey AS, Stevens LA, Schmid CH, Zhang YL, Castro AF 3rd, Feldman HI, et al. A new equation to estimate glomerular filtration rate. Ann Intern Med. 2009;150(9):604–12. doi: 10.7326/0003-4819-150-9-200905050-00006 19414839 PMC2763564

[17] Koetsier M, Lutgers HL, de Jonge C, Links TP, Smit AJ, Graaff R. Reference values of skin autofluorescence. Diabetes Technol Ther. 2010;12(5):399–403. doi: 10.1089/dia.2009.0113 20388050

[18] Raggi P, Cooil B, Callister TQ. Use of electron beam tomography data to develop models for prediction of hard coronary events. Am Heart J. 2001;141(3):375–82. doi: 10.1067/mhj.2001.113220 11231434

[19] Goff DC, Jr., Lloyd-Jones DM, Bennett G, Coady S, D’Agostino RB, Gibbons R, et al. 2013 ACC/AHA guideline on the assessment of cardiovascular risk: a report of the American College of Cardiology/American Heart Association Task Force on Practice Guidelines. Circulation. 2014;129(25 Suppl 2):S49–73. doi: 10.1161/01.cir.0000437741.48606.98 24222018

[20] Singh R, Barden A, Mori T, Beilin L. Advanced glycation end-products: a review. Diabetologia. 2001;44(2):129–46. doi: 10.1007/s001250051591 11270668

[21] Budoff MJ, Achenbach S, Blumenthal RS, Carr JJ, Goldin JG, Greenland P, et al. Assessment of coronary artery disease by cardiac computed tomography: a scientific statement from the American Heart Association Committee on Cardiovascular Imaging and Intervention, Council on Cardiovascular Radiology and Intervention, and Committee on Cardiac Imaging, Council on Clinical Cardiology. Circulation. 2006;114(16):1761–91. doi: 10.1161/CIRCULATIONAHA.106.178458 17015792

[22] Meerwaldt R, Lutgers HL, Links TP, Graaff R, Baynes JW, Gans RO, et al. Skin autofluorescence is a strong predictor of cardiac mortality in diabetes. Diabetes Care. 2007;30(1):107–12. doi: 10.2337/dc06-1391 17192342

[23] Smilowitz NR, Sampson BA, Abrecht CR, Siegfried JS, Hochman JS, Reynolds HR. Women have less severe and extensive coronary atherosclerosis in fatal cases of ischemic heart disease: an autopsy study. Am Heart J. 2011;161(4):681–8. doi: 10.1016/j.ahj.2010.12.022 21473966

[24] Mulder DJ, Water TV, Lutgers HL, Graaff R, Gans RO, Zijlstra F, et al. Skin autofluorescence, a novel marker for glycemic and oxidative stress-derived advanced glycation endproducts: an overview of current clinical studies, evidence, and limitations. Diabetes Technol Ther. 2006;8(5):523–35. doi: 10.1089/dia.2006.8.523 17037967

