# Supplementary material for: Association between skin autofluorescence and coronary calcification in the general population
Source: PLoS One. 2024 Aug 26;19(8):e0309059. doi: 10.1371/journal.pone.0309059 (PMC11346912; doi:10.1371/journal.pone.0309059)
Supplement: S1 Table — (DOCX) [file pone.0309059.s001.docx]

**Appendices**

**S1 Table.** Odds ratios for the presence of coronary artery calcium by 1 unit increase of skin autofluorescence by sex

|  | CACS > 0 | |  |
| --- | --- | --- | --- |
|  | Odds ratio | 95%CI | P value |
| Men (n=1629) |  |  |  |
| Model 1 | 2.66 | (2.00, 3.58) | <0.001 |
| Model 2 | 1.35 | (0.98, 1.85) | 0.065 |
| Model 3 | 1.20 | (0.87, 1.68) | 0.275 |
|  |  |  |  |
| Women (n=2210) |  |  |  |
| Model 1 | 2.81 | (2.23, 3.55) | <0.001 |
| Model 2 | 1.37 | (1.06, 1.77) | 0.017 |
| Model 3 | 1.18 | (0.90, 1.54) | 0.237 |

CACS: coronary artery calcium score; CI: confidence interval

Reference group: coronary artery calcium score equals to zero

Model 1: univariate logistic regression modelling of skin autofluorescence

Model 2: adjusted for age

Model 3: model 2 plus additional adjustments for smoking status, total cholesterol, high-density lipoprotein cholesterol, systolic blood pressure
