# Supplementary material for: Association between skin autofluorescence and coronary calcification in the general population
Source: PLoS One. 2024 Aug 26;19(8):e0309059. doi: 10.1371/journal.pone.0309059 (PMC11346912; doi:10.1371/journal.pone.0309059)
Supplement: Attachment — Submitted filename: EB Response to Reviewers.docx [file pone.0309059.s002.docx]

Point by point response to reviewers

Dear editor

We would like to thank you for your decision dated 20 may 2024 and the opportunity to resubmit a revised version of our manuscript entitled ‘Association between skin autofluorescence and coronary calcification in the general population’. We would also like to thank the reviewers for their careful review and constructive comments on our manuscript.

In the revised manuscript, we have considered the comments and suggestions outlined by the edition and reviewers. The manuscript is modified accordingly. We have also answered the specific questions which were posed, and our response in a point-by-point manner is provided below.

We hope that in its present form, our manuscript can be accepted for publication.

Sincerely, on behalf of all co-authors,

H. Erik Boersma, MD

Bruce H.R. Wolffenbuttel, MD, PhD

Journal Requirements:

1. When submitting your revision, we need you to address these additional requirements.

Please ensure that your manuscript meets PLOS ONE's style requirements, including those for file naming. The PLOS ONE style templates can be found at 
<https://journals.plos.org/plosone/s/file?id=wjVg/PLOSOne_formatting_sample_main_body.pdf> and 
<https://journals.plos.org/plosone/s/file?id=ba62/PLOSOne_formatting_sample_title_authors_affiliations.pdf>.

- The manuscript was adapted to meet PLOS ONE's style requirements.

2. Thank you for stating the following in the Competing Interests section: 
[I have read the journal's policy and the authors of this manuscript have the following competing interests:
A.J. Smit is founder and shareholder in Diagnoptics Technologies (Groningen, the Netherlands), manufacturer of the AGE reader that was used in the present study. M. Oudkerk is involved in the company iDNA BV. M. Vonder is a consultant for iDNA BV. All other authors declare that there is no duality of interest associated with this manuscript.].

Please confirm that this does not alter your adherence to all PLOS ONE policies on sharing data and materials, by including the following statement: ""This does not alter our adherence to PLOS ONE policies on sharing data and materials.” (as detailed online in our guide for authors <http://journals.plos.org/plosone/s/competing-interests>).  If there are restrictions on sharing of data and/or materials, please state these. Please note that we cannot proceed with consideration of your article until this information has been declared.

Please include your updated Competing Interests statement in your cover letter; we will change the online submission form on your behalf.

- Adapted competing interest statement:

I have read the journal's policy and the authors of this manuscript have the following competing interests:
A.J. Smit is founder and shareholder in Diagnoptics Technologies (Groningen, the Netherlands), manufacturer of the AGE reader that was used in the present study. M. Oudkerk is involved in the company iDNA BV. M. Vonder is a consultant for iDNA BV. All other authors declare that there is no duality of interest associated with this manuscript. This does not alter our adherence to PLOS ONE policies on sharing data and materials.

3. We note that you have indicated that there are restrictions to data sharing for this study. PLOS only allows data to be available upon request if there are legal or ethical restrictions on sharing data publicly. For more information on unacceptable data access restrictions, please see <http://journals.plos.org/plosone/s/data-availability#loc-unacceptable-data-access-restrictions>.

Before we proceed with your manuscript, please address the following prompts:

a) If there are ethical or legal restrictions on sharing a de-identified data set, please explain them in detail (e.g., data contain potentially identifying or sensitive patient information, data are owned by a third-party organization, etc.) and who has imposed them (e.g., a Research Ethics Committee or Institutional Review Board, etc.). Please also provide contact information for a data access committee, ethics committee, or other institutional body to which data requests may be sent.

b) If there are no restrictions, please upload the minimal anonymized data set necessary to replicate your study findings to a stable, public repository and provide us with the relevant URLs, DOIs, or accession numbers. For a list of recommended repositories, please see
<https://journals.plos.org/plosone/s/recommended-repositories>. You also have the option of uploading the data as Supporting Information files, but we would recommend depositing data directly to a data repository if possible.

We will update your Data Availability statement on your behalf to reflect the information you provide.

- Adapted data availability statement:

Data cannot be shared publicly because of ethical restrictions imposed by the Lifelines Scientific Board and the Medical Ethical Committee of the University Medical Center Groningen related to protecting participant privacy. Data are available from the Lifelines research office ([research@lifelines.nl](mailto:research@lifelines.nl)). All international researchers can obtain data at the Lifelines research office, for which a fee is required. The Lifelines system allows access for reproducibility of the study results.

4. Please include captions for your Supporting Information files at the end of your manuscript, and update any in-text citations to match accordingly. Please see our Supporting Information guidelines for more information: <http://journals.plos.org/plosone/s/supporting-information>.

- Captions for Supporting Information were added at the end of the manuscript.

5. Please review your reference list to ensure that it is complete and correct. If you have cited papers that have been retracted, please include the rationale for doing so in the manuscript text, or remove these references and replace them with relevant current references. Any changes to the reference list should be mentioned in the rebuttal letter that accompanies your revised manuscript. If you need to cite a retracted article, indicate the article’s retracted status in the References list and also include a citation and full reference for the retraction notice.

- We reviewed the reference list; no retracted papers were cited. A segment about skin **phototypes, as suggested by a reviewer, was added to the manuscript with accompanying added reference.**

**Review Comments to the Author**

**Reviewer #1: This study appears to have been conducted in appropriate way. The rationale of the study is well-justified, clinical relevance also is beyond doubt. Research approach, including data collection methods, whole data analysis was described in sufficient details. Ethical considerations are addressed. Findings of this study could help improve clinical practice, especially in the field of primary prevention (risk stratification management). This is very important, because progress in risk stratification strategies can improve patient outcomes by providing more effective goal-oriented medical care and what is not less important reduce healthcare costs. Authors have demonstrated a thoughtful and rigorous approach to the research process.**

- We thank the reviewer for his / her comments and evaluation.

**Reviewer #2: The paper is overall well written and figures out an interesting association among a putative cross talk between simple skin features and cardiovascular risk. Since I feel the paper could represent an innovative evidence on the issue, I would suggest some revisions.**

**A) A well known limitation of Advanced Glycation End Products (AGEs) method is that its quantification could be interfered by skin pigments, such as melanin, which limits its application on patients with a darker skin tone (1). To achieve a stronger statistical strenght, I would suggest the authors to explain in the RESULTS section the different phototypes of the analyzed population and perform stratification (eg. multivariate analysis) on this data.**

**(1) Jaisson, S.; Gillery, P. Methods to assess advanced glycation end-products. Curr. Opin. Clin. Nutr. Metab. Care 2021, 24, 411–415.**

- We thank the reviewer for his / her comment and have adapted the manuscript, line 391-395. We omitted extra multivariate analysis on different skin photo types, as the vast majority of Lifelines participants have a Caucasian ethnicity (>98%), therefore influence of skin pigmentation differences is very limited.

**B) There is already evidence in the literature that AGEs and antibodies against AGE have been explored in some chronic inflammatory skin diseases, such as psoriasis and hidradenitis suppurativa, which are in themselves associated with greater cardiovascular risk (2) (3) I would suggest broadening the discussion by reporting this data and reasoning on how cardiovascular risk of such patients may be linked to the demonstrated association they have with coronary calcification.**

**(2) Papagrigoraki A, Maurelli M, Del Giglio M, Gisondi P, Girolomoni G. Advanced Glycation End Products in the Pathogenesis of Psoriasis. Int J Mol Sci. 2017 Nov 20;18(11):2471. doi: 10.3390/ijms18112471.**
**(3) Macchiarella G, Cornacchione V, Cojean C, Riker J, Wang Y, Te H, Ceci M, Gudjonsson JE, Gaulis S, Goetschy JF, Wollschlegel A, Gass SK, Oetliker-Contin S, Wettstein-Ling B, Schaefer DJ, Meschberger P, de Roche R, Osinga R, Wieczorek G, Naumann U, Lehmann JCU, Schubart A, Hofmann A, Roth L, Florencia EF, Loesche C, Traggiai E, Avrameas A, Prens EP, Röhn TA, Roediger B. Disease Association of Anti‒Carboxyethyl Lysine Autoantibodies in Hidradenitis Suppurativa. J Invest Dermatol. 2023 Feb;143(2):273-283.e12. doi: 10.1016/j.jid.2022.08.051.**

- In our manuscript we comment on the involvement of AGEs in atherosclerosis-related vascular damage as well as the interaction of AGEs with RAGE in vascular disease. Systemic autoimmune diseases in general are associated with inflammation, and oxidative stress promoting the formation of advanced glycation endproducts (4). As this is not specific for inflammatory skin diseases, we feel broadening the discussion on chronic inflammatory skin diseases is only relevant for very specific patient populations. Additionally, in the Lifelines cohort study skin conditions are self-reported, limiting the reliability of a potential subgroup analysis. We appreciate the reviewer’s suggestions.

(4) Reynaert NL, Gopal P, Rutten EPA, Wouters EFM, Schalkwijk CG. Advanced glycation end products and their receptor in age-related, non-communicable chronic inflammatory diseases; Overview of clinical evidence and potential contributions to disease. Int J Biochem Cell Biol. 2016 Dec;81(Pt B):403-418. doi: 10.1016/j.biocel.2016.06.016.

We thank the reviewer for his / her comments and favorable evaluation.
