# Supplementary material for: Association between skin autofluorescence and coronary calcification in the general population
Source: PLoS One. 2024 Aug 26;19(8):e0309059. doi: 10.1371/journal.pone.0309059 (PMC11346912; doi:10.1371/journal.pone.0309059)
Supplement: Peer review history

# Peer review: Decision Letter 0

**DOI:** 10.1371/journal.pone.0309059.r001

**Authors:** Vincenzo Lionetti

20 May 2024

PONE-D-24-04875Association between skin autofluorescence and coronary calcification in the general populationPLOS ONE

Dear Dr. Wolffenbuttel,

Thank you for submitting your manuscript to PLOS ONE. After careful consideration, we feel that it has merit but does not fully meet PLOS ONE’s publication criteria as it currently stands. Therefore, we invite you to submit a revised version of the manuscript that addresses the points raised during the review process.

==============================

**ACADEMIC EDITOR: All issues raised by expert reviewers are required. Please improve the discussion by limiting speculations. ==============================**

**Please submit your revised manuscript by Jul 04 2024 11:59PM. If you will need more time than this to complete your revisions, please reply to this message or contact the journal office at plosone@plos.org. When you're ready to submit your revision, log on to https://www.editorialmanager.com/pone/ and select the 'Submissions Needing Revision' folder to locate your manuscript file.**

**Please include the following items when submitting your revised manuscript:**

- **A rebuttal letter that responds to each point raised by the academic editor and reviewer(s). You should upload this letter as a separate file labeled 'Response to Reviewers'.**
- **A marked-up copy of your manuscript that highlights changes made to the original version. You should upload this as a separate file labeled 'Revised Manuscript with Track Changes'.**
- **An unmarked version of your revised paper without tracked changes. You should upload this as a separate file labeled 'Manuscript'.**

**If you would like to make changes to your financial disclosure, please include your updated statement in your cover letter. Guidelines for resubmitting your figure files are available below the reviewer comments at the end of this letter**.

**If applicable, we recommend that you deposit your laboratory protocols in protocols.io to enhance the reproducibility of your results. Protocols.io assigns your protocol its own identifier (DOI) so that it can be cited independently in the future. For instructions see: https://journals.plos.org/plosone/s/submission-guidelines#loc-laboratory-protocols. Additionally, PLOS ONE offers an option for publishing peer-reviewed Lab Protocol articles, which describe protocols hosted on protocols.io. Read more information on sharing protocols at https://plos.org/protocols?utm_medium=editorial-email&utm_source=authorletters&utm_campaign=protocols**.

**We look forward to receiving your revised manuscript.**

**Kind regards,**

**Vincenzo Lionetti, M.D., PhD**

**Academic Editor**

**PLOS ONE**

Journal Requirements:

1. When submitting your revision, we need you to address these additional requirements.

Please ensure that your manuscript meets PLOS ONE's style requirements, including those for file naming. The PLOS ONE style templates can be found at 

https://journals.plos.org/plosone/s/file?id=wjVg/PLOSOne_formatting_sample_main_body.pdf and 

https://journals.plos.org/plosone/s/file?id=ba62/PLOSOne_formatting_sample_title_authors_affiliations.pdf.

2. Thank you for stating the following in the Competing Interests section: 

[I have read the journal's policy and the authors of this manuscript have the following competing interests:

A.J. Smit is founder and shareholder in Diagnoptics Technologies (Groningen, the Netherlands), manufacturer of the AGE reader that was used in the present study. M. Oudkerk is involved in the company iDNA BV. M. Vonder is a consultant for iDNA BV. All other authors declare that there is no duality of interest associated with this manuscript.]. 

Please confirm that this does not alter your adherence to all PLOS ONE policies on sharing data and materials, by including the following statement: ""This does not alter our adherence to  PLOS ONE policies on sharing data and materials.” (as detailed online in our guide for authors http://journals.plos.org/plosone/s/competing-interests).  If there are restrictions on sharing of data and/or materials, please state these. Please note that we cannot proceed with consideration of your article until this information has been declared. 

Please include your updated Competing Interests statement in your cover letter; we will change the online submission form on your behalf.

3. We note that you have indicated that there are restrictions to data sharing for this study. PLOS only allows data to be available upon request if there are legal or ethical restrictions on sharing data publicly. For more information on unacceptable data access restrictions, please see http://journals.plos.org/plosone/s/data-availability#loc-unacceptable-data-access-restrictions. 

Before we proceed with your manuscript, please address the following prompts:

a) If there are ethical or legal restrictions on sharing a de-identified data set, please explain them in detail (e.g., data contain potentially identifying or sensitive patient information, data are owned by a third-party organization, etc.) and who has imposed them (e.g., a Research Ethics Committee or Institutional Review Board, etc.). Please also provide contact information for a data access committee, ethics committee, or other institutional body to which data requests may be sent.

b) If there are no restrictions, please upload the minimal anonymized data set necessary to replicate your study findings to a stable, public repository and provide us with the relevant URLs, DOIs, or accession numbers. For a list of recommended repositories, please see

https://journals.plos.org/plosone/s/recommended-repositories. You also have the option of uploading the data as Supporting Information files, but we would recommend depositing data directly to a data repository if possible.

We will update your Data Availability statement on your behalf to reflect the information you provide.

4. Please include captions for your Supporting Information files at the end of your manuscript, and update any in-text citations to match accordingly. Please see our Supporting Information guidelines for more information: http://journals.plos.org/plosone/s/supporting-information. 

**5. Please review your reference list to ensure that it is complete and correct. If you have cited papers that have been retracted, please include the rationale for doing so in the manuscript text, or remove these references and replace them with relevant current references. Any changes to the reference list should be mentioned in the rebuttal letter that accompanies your revised manuscript. If you need to cite a retracted article, indicate the article’s retracted status in the References list and also include a citation and full reference for the retraction notice**.

**[Note: HTML markup is below. Please do not edit.]**

**Reviewers' comments**:

**Reviewer's Responses to Questions**

**Comments to the Author**

**1. Is the manuscript technically sound, and do the data support the conclusions?**

**The manuscript must describe a technically sound piece of scientific research with data that supports the conclusions. Experiments must have been conducted rigorously, with appropriate controls, replication, and sample sizes. The conclusions must be drawn appropriately based on the data presented.**

**Reviewer #1: Yes**

**Reviewer #2: Partly**

**********

**2. Has the statistical analysis been performed appropriately and rigorously?**

**Reviewer #1: Yes**

**Reviewer #2: Yes**

**********

**3. Have the authors made all data underlying the findings in their manuscript fully available?**

**The PLOS Data policy requires authors to make all data underlying the findings described in their manuscript fully available without restriction, with rare exception (please refer to the Data Availability Statement in the manuscript PDF file). The data should be provided as part of the manuscript or its supporting information, or deposited to a public repository. For example, in addition to summary statistics, the data points behind means, medians and variance measures should be available. If there are restrictions on publicly sharing data—e.g. participant privacy or use of data from a third party—those must be specified.**

**Reviewer #1: Yes**

**Reviewer #2: Yes**

**********

**4. Is the manuscript presented in an intelligible fashion and written in standard English?**

**PLOS ONE does not copyedit accepted manuscripts, so the language in submitted articles must be clear, correct, and unambiguous. Any typographical or grammatical errors should be corrected at revision, so please note any specific errors here.**

**Reviewer #1: Yes**

**Reviewer #2: Yes**

**********

**5. Review Comments to the Author**

**Please use the space provided to explain your answers to the questions above. You may also include additional comments for the author, including concerns about dual publication, research ethics, or publication ethics. (Please upload your review as an attachment if it exceeds 20,000 characters)**

**Reviewer #1: This study appears to have been conducted in appropriate way. The rationale of the study is well-justified, clinical relevance also is beyond doubt. Research approach, including data collection methods, whole data analysis was described in sufficient details. Ethical considerations are addressed. Findings of this study could help improve clinical practice, especially in the field of primary prevention (risk stratification management). This is very important, because progress in risk stratification strategies can improve patient outcomes by providing more effective goal-oriented medical care and what is not less important reduce healthcare costs. Authors have demonstrated a thoughtful and rigorous approach to the research process.**

**Reviewer #2: The paper is overall well written and figures out an interesting association among a putative cross talk between simple skin features and cardiovascular risk. Since I feel the paper could represent an innovative evidence on the issue, I would suggest some revisions**.

**A) A well known limitation of Advanced Glycation End Products (AGEs) method is that its quantification could be interfered by skin pigments, such as melanin, which limits its application on patients with a darker skin tone (1). To achieve a stronger statistical strenght, I would suggest the authors to explain in the RESULTS section the different phototypes of the analyzed population and perform stratification (eg. multivariate analysis) on this data**.

**(1) Jaisson, S.; Gillery, P. Methods to assess advanced glycation end-products. Curr. Opin. Clin. Nutr. Metab. Care 2021, 24, 411–415**.

**B) There is already evidence in the literature that AGEs and antibodies against AGE have been explored in some chronic inflammatory skin diseases, such as psoriasis and hidradenitis suppurativa, which are in themselves associated with greater cardiovascular risk (2) (3) I would suggest broadening the discussion by reporting this data and reasoning on how cardiovascular risk of such patients may be linked to the demonstrated association they have with coronary calcification**.

**(2) Papagrigoraki A, Maurelli M, Del Giglio M, Gisondi P, Girolomoni G. Advanced Glycation End Products in the Pathogenesis of Psoriasis. Int J Mol Sci. 2017 Nov 20;18(11):2471. doi: 10.3390/ijms18112471**.

**(3) Macchiarella G, Cornacchione V, Cojean C, Riker J, Wang Y, Te H, Ceci M, Gudjonsson JE, Gaulis S, Goetschy JF, Wollschlegel A, Gass SK, Oetliker-Contin S, Wettstein-Ling B, Schaefer DJ, Meschberger P, de Roche R, Osinga R, Wieczorek G, Naumann U, Lehmann JCU, Schubart A, Hofmann A, Roth L, Florencia EF, Loesche C, Traggiai E, Avrameas A, Prens EP, Röhn TA, Roediger B. Disease Association of Anti‒Carboxyethyl Lysine Autoantibodies in Hidradenitis Suppurativa. J Invest Dermatol. 2023 Feb;143(2):273-283.e12. doi: 10.1016/j.jid.2022.08.051.**

**********

**6. PLOS authors have the option to publish the peer review history of their article (what does this mean?). If published, this will include your full peer review and any attached files**.

If you choose “no”, your identity will remain anonymous but your review may still be made public.

**Do you want your identity to be public for this peer review? For information about this choice, including consent withdrawal, please see our Privacy Policy.**

**Reviewer #1: No**

**Reviewer #2: No**

**********

**[NOTE: If reviewer comments were submitted as an attachment file, they will be attached to this email and accessible via the submission site. Please log into your account, locate the manuscript record, and check for the action link "View Attachments". If this link does not appear, there are no attachment files.]**

**While revising your submission, please upload your figure files to the Preflight Analysis and Conversion Engine (PACE) digital diagnostic tool, https://pacev2.apexcovantage.com/. PACE helps ensure that figures meet PLOS requirements. To use PACE, you must first register as a user. Registration is free. Then, login and navigate to the UPLOAD tab, where you will find detailed instructions on how to use the tool. If you encounter any issues or have any questions when using PACE, please email PLOS at figures@plos.org. Please note that Supporting Information files do not need this step.**

# Author comment: Author response to Decision Letter 0

**DOI:** 10.1371/journal.pone.0309059.r002

25 Jul 2024

Journal Requirements:

1. When submitting your revision, we need you to address these additional requirements.

Please ensure that your manuscript meets PLOS ONE's style requirements, including those for file naming. The PLOS ONE style templates can be found at 

https://journals.plos.org/plosone/s/file?id=wjVg/PLOSOne_formatting_sample_main_body.pdf and 

https://journals.plos.org/plosone/s/file?id=ba62/PLOSOne_formatting_sample_title_authors_affiliations.pdf.

- The manuscript was adapted to meet PLOS ONE's style requirements.

2. Thank you for stating the following in the Competing Interests section: 

[I have read the journal's policy and the authors of this manuscript have the following competing interests:

A.J. Smit is founder and shareholder in Diagnoptics Technologies (Groningen, the Netherlands), manufacturer of the AGE reader that was used in the present study. M. Oudkerk is involved in the company iDNA BV. M. Vonder is a consultant for iDNA BV. All other authors declare that there is no duality of interest associated with this manuscript.]. 

Please confirm that this does not alter your adherence to all PLOS ONE policies on sharing data and materials, by including the following statement: ""This does not alter our adherence to PLOS ONE policies on sharing data and materials.” (as detailed online in our guide for authors http://journals.plos.org/plosone/s/competing-interests). If there are restrictions on sharing of data and/or materials, please state these. Please note that we cannot proceed with consideration of your article until this information has been declared. 

Please include your updated Competing Interests statement in your cover letter; we will change the online submission form on your behalf.

- Adapted competing interest statement:

I have read the journal's policy and the authors of this manuscript have the following competing interests:

A.J. Smit is founder and shareholder in Diagnoptics Technologies (Groningen, the Netherlands), manufacturer of the AGE reader that was used in the present study. M. Oudkerk is involved in the company iDNA BV. M. Vonder is a consultant for iDNA BV. All other authors declare that there is no duality of interest associated with this manuscript. This does not alter our adherence to PLOS ONE policies on sharing data and materials.

3. We note that you have indicated that there are restrictions to data sharing for this study. PLOS only allows data to be available upon request if there are legal or ethical restrictions on sharing data publicly. For more information on unacceptable data access restrictions, please see http://journals.plos.org/plosone/s/data-availability#loc-unacceptable-data-access-restrictions. 

Before we proceed with your manuscript, please address the following prompts:

a) If there are ethical or legal restrictions on sharing a de-identified data set, please explain them in detail (e.g., data contain potentially identifying or sensitive patient information, data are owned by a third-party organization, etc.) and who has imposed them (e.g., a Research Ethics Committee or Institutional Review Board, etc.). Please also provide contact information for a data access committee, ethics committee, or other institutional body to which data requests may be sent.

b) If there are no restrictions, please upload the minimal anonymized data set necessary to replicate your study findings to a stable, public repository and provide us with the relevant URLs, DOIs, or accession numbers. For a list of recommended repositories, please see

https://journals.plos.org/plosone/s/recommended-repositories. You also have the option of uploading the data as Supporting Information files, but we would recommend depositing data directly to a data repository if possible.

We will update your Data Availability statement on your behalf to reflect the information you provide.

- Adapted data availability statement:

Data cannot be shared publicly because of ethical restrictions imposed by the Lifelines Scientific Board and the Medical Ethical Committee of the University Medical Center Groningen related to protecting participant privacy. Data are available from the Lifelines research office (research@lifelines.nl). All international researchers can obtain data at the Lifelines research office, for which a fee is required. The Lifelines system allows access for reproducibility of the study results.

4. Please include captions for your Supporting Information files at the end of your manuscript, and update any in-text citations to match accordingly. Please see our Supporting Information guidelines for more information: http://journals.plos.org/plosone/s/supporting-information. 

- Captions for Supporting Information were added at the end of the manuscript.

5. Please review your reference list to ensure that it is complete and correct. If you have cited papers that have been retracted, please include the rationale for doing so in the manuscript text, or remove these references and replace them with relevant current references. Any changes to the reference list should be mentioned in the rebuttal letter that accompanies your revised manuscript. If you need to cite a retracted article, indicate the article’s retracted status in the References list and also include a citation and full reference for the retraction notice.

- We reviewed the reference list; no retracted papers were cited. A segment about skin phototypes, as suggested by a reviewer, was added to the manuscript with accompanying added reference.

Review Comments to the Author

Reviewer #1: This study appears to have been conducted in appropriate way. The rationale of the study is well-justified, clinical relevance also is beyond doubt. Research approach, including data collection methods, whole data analysis was described in sufficient details. Ethical considerations are addressed. Findings of this study could help improve clinical practice, especially in the field of primary prevention (risk stratification management). This is very important, because progress in risk stratification strategies can improve patient outcomes by providing more effective goal-oriented medical care and what is not less important reduce healthcare costs. Authors have demonstrated a thoughtful and rigorous approach to the research process.

- We thank the reviewer for his / her comments and evaluation.

Reviewer #2: The paper is overall well written and figures out an interesting association among a putative cross talk between simple skin features and cardiovascular risk. Since I feel the paper could represent an innovative evidence on the issue, I would suggest some revisions.

A) A well known limitation of Advanced Glycation End Products (AGEs) method is that its quantification could be interfered by skin pigments, such as melanin, which limits its application on patients with a darker skin tone (1). To achieve a stronger statistical strenght, I would suggest the authors to explain in the RESULTS section the different phototypes of the analyzed population and perform stratification (eg. multivariate analysis) on this data.

(1) Jaisson, S.; Gillery, P. Methods to assess advanced glycation end-products. Curr. Opin. Clin. Nutr. Metab. Care 2021, 24, 411–415.

- We thank the reviewer for his / her comment and have adapted the manuscript, line 391-395. We omitted extra multivariate analysis on different skin photo types, as the vast majority of Lifelines participants have a Caucasian ethnicity (>98%), therefore influence of skin pigmentation differences is very limited.

B) There is already evidence in the literature that AGEs and antibodies against AGE have been explored in some chronic inflammatory skin diseases, such as psoriasis and hidradenitis suppurativa, which are in themselves associated with greater cardiovascular risk (2) (3) I would suggest broadening the discussion by reporting this data and reasoning on how cardiovascular risk of such patients may be linked to the demonstrated association they have with coronary calcification.

(2) Papagrigoraki A, Maurelli M, Del Giglio M, Gisondi P, Girolomoni G. Advanced Glycation End Products in the Pathogenesis of Psoriasis. Int J Mol Sci. 2017 Nov 20;18(11):2471. doi: 10.3390/ijms18112471.

(3) Macchiarella G, Cornacchione V, Cojean C, Riker J, Wang Y, Te H, Ceci M, Gudjonsson JE, Gaulis S, Goetschy JF, Wollschlegel A, Gass SK, Oetliker-Contin S, Wettstein-Ling B, Schaefer DJ, Meschberger P, de Roche R, Osinga R, Wieczorek G, Naumann U, Lehmann JCU, Schubart A, Hofmann A, Roth L, Florencia EF, Loesche C, Traggiai E, Avrameas A, Prens EP, Röhn TA, Roediger B. Disease Association of Anti‒Carboxyethyl Lysine Autoantibodies in Hidradenitis Suppurativa. J Invest Dermatol. 2023 Feb;143(2):273-283.e12. doi: 10.1016/j.jid.2022.08.051.

- In our manuscript we comment on the involvement of AGEs in atherosclerosis-related vascular damage as well as the interaction of AGEs with RAGE in vascular disease. Systemic autoimmune diseases in general are associated with inflammation, and oxidative stress promoting the formation of advanced glycation endproducts (4). As this is not specific for inflammatory skin diseases, we feel broadening the discussion on chronic inflammatory skin diseases is only relevant for very specific patient populations. Additionally, in the Lifelines cohort study skin conditions are self-reported, limiting the reliability of a potential subgroup analysis. We appreciate the reviewer’s suggestions. 

(4) Reynaert NL, Gopal P, Rutten EPA, Wouters EFM, Schalkwijk CG. Advanced glycation end products and their receptor in age-related, non-communicable chronic inflammatory diseases; Overview of clinical evidence and potential contributions to disease. Int J Biochem Cell Biol. 2016 Dec;81(Pt B):403-418. doi: 10.1016/j.biocel.2016.06.016. 

We thank the reviewer for his / her comments and favorable evaluation.

Attachment Submitted filename: EB Response to Reviewers.docx

# Editor report: Decision Letter 1

**DOI:** 10.1371/journal.pone.0309059.r003

**Authors:** Vincenzo Lionetti

6 Aug 2024

Association between skin autofluorescence and coronary calcification in the general population

PONE-D-24-04875R1

Dear Dr. Wolffenbuttel,

We’re pleased to inform you that your manuscript has been judged scientifically suitable for publication and will be formally accepted for publication once it meets all outstanding technical requirements.

Within one week, you’ll receive an e-mail detailing the required amendments. When these have been addressed, you’ll receive a formal acceptance letter and your manuscript will be scheduled for publication.

An invoice will be generated when your article is formally accepted. Please note, if your institution has a publishing partnership with PLOS and your article meets the relevant criteria, all or part of your publication costs will be covered. Please make sure your user information is up-to-date by logging into Editorial Manager at Editorial Manager® and clicking the ‘Update My Information' link at the top of the page. If you have any questions relating to publication charges, please contact our Author Billing department directly at authorbilling@plos.org.

If your institution or institutions have a press office, please notify them about your upcoming paper to help maximize its impact. If they’ll be preparing press materials, please inform our press team as soon as possible -- no later than 48 hours after receiving the formal acceptance. Your manuscript will remain under strict press embargo until 2 pm Eastern Time on the date of publication. For more information, please contact onepress@plos.org.

Kind regards,

Vincenzo Lionetti, M.D., PhD

Academic Editor

PLOS ONE

Additional Editor Comments (optional):

Reviewers' comments:

# Editor report: Acceptance letter

**DOI:** 10.1371/journal.pone.0309059.r004

**Authors:** Vincenzo Lionetti

15 Aug 2024

PONE-D-24-04875R1 

PLOS ONE

Dear Dr. Wolffenbuttel, 

I'm pleased to inform you that your manuscript has been deemed suitable for publication in PLOS ONE. Congratulations! Your manuscript is now being handed over to our production team.

At this stage, our production department will prepare your paper for publication. This includes ensuring the following:

* All references, tables, and figures are properly cited

* All relevant supporting information is included in the manuscript submission,

* There are no issues that prevent the paper from being properly typeset

If revisions are needed, the production department will contact you directly to resolve them. If no revisions are needed, you will receive an email when the publication date has been set. At this time, we do not offer pre-publication proofs to authors during production of the accepted work. Please keep in mind that we are working through a large volume of accepted articles, so please give us a few weeks to review your paper and let you know the next and final steps. 

Lastly, if your institution or institutions have a press office, please let them know about your upcoming paper now to help maximize its impact. If they'll be preparing press materials, please inform our press team within the next 48 hours. Your manuscript will remain under strict press embargo until 2 pm Eastern Time on the date of publication. For more information, please contact onepress@plos.org.

If we can help with anything else, please email us at customercare@plos.org.

Thank you for submitting your work to PLOS ONE and supporting open access. 

Kind regards, 

PLOS ONE Editorial Office Staff

on behalf of

Prof. Vincenzo Lionetti 

Academic Editor

PLOS ONE
